# Performance of UNHCR nutrition programs in post-emergency refugee camps

**DOI:** 10.1186/1752-1505-5-23

**Published:** 2011-10-26

**Authors:** Shannon Doocy, Hannah Tappis, Christopher Haskew, Caroline Wilkinson, Paul Spiegel

**Affiliations:** 1 Bloomberg School of Public Health, Johns Hopkins University, Baltimore, Maryland, USA; 2 United Nations High Commissioner for Refugees, Geneva, Switzerland

## Abstract

**Background:**

The United Nations High Commissioner for Refugees (UNHCR) launched a health information system (HIS) in 2005 to enhance quality and consistency of routine health information available in post-emergency refugee camps. This paper reviews nutrition indicators and examines their application for monitoring and evaluating the performance of UNHCR nutrition programs in more than 90 refugee camps in 18 countries.

**Methods:**

The HIS is a primary source of feeding program data which is collected using standardized case definitions and reporting formats across refugee camps in multiple settings. Data was aggregated across time periods and within and across countries for analysis. Basic descriptive statistics were then compared to UNHCR program performance standards.

**Results:**

Camp populations covered by the HIS ranged from 192,000 to 219,000 between 2007 and mid-2009; 87% of under five children covered by the HIS were in Africa and 13% in Asia. Average moderate acute malnutrition (MAM) and severe acute malnutrition (SAM) rates reported in 74 of 81 camps for the 2007-2009 time periods were 7.0% and 1.6%, respectively. The supplementary feeding program (SFP) admission rate was 151/1000/yr with 93% of SFP admissions occurring in Africa. SFP performance consistently exceeded all UNHCR standards with the exception of length of enrollment. Average length of SFP enrollment was 12 weeks in Africa and 22 weeks in Asia as compared to the UNHCR standard of < 8 weeks. The therapeutic feeding program (TFP) admission was 22/1000/yr with 95% of TFP admissions in Africa. TFP performance met UNHCR standards with the exception of daily weight gain.

**Conclusions:**

Inclusion of children identified as moderately and severely wasted in the HIS would allow UNHCR to better track and respond to changes in nutrition status. Improved growth monitoring coverage or active malnutrition surveillance would increase UNHCR's ability to identify and treat cases of acute malnutrition. Expansion of nutrition reporting to address the transition to community-based therapeutic care is essential for adequate performance monitoring in the future. In terms of program priorities, a focus on camps and countries with large refugee populations and high feeding program enrollment rates would have the greatest impact in terms of absolute reductions in the incidence and prevalence of malnutrition.

## Introduction

In 2009, there were an estimated 10.5 million refugees of concern to the United Nations High Commissioner of Refugees (UNHCR) [1]. Refugees represent a minority of the displaced population worldwide and less than half of refugees live in camps. A small proportion live among rural host communities and the majority are integrated among host country urban populations [2,3] UNHCR refugee camps are predominantly in rural areas of Africa and Asia in protracted or post-emergency contexts where their populations benefit from relatively stable environments and low mortality rates. In contrast to acute phase emergencies, mortality rates rarely exceed emergency thresholds and are often lower among refugees as compared to surrounding host populations [4] Factors contributing to lower mortality include better access to primary health care, water and sanitation, food rations, and public health measures such as disease surveillance and response [3,5,6]. Malnutrition contributes to more than half of child deaths in less-developed settings and the association between malnutrition and mortality in refugee camp settings is well documented [7,8]. However, as a result of emergency nutrition programming that includes routine distribution of food rations, identification of malnourished children through screening and growth monitoring, and rehabilitation of malnourished individuals through supplementary and therapeutic feeding programs, malnutrition is no longer a major a cause of mortality in refugee camp settings. In UNHCR camps, the World Food Program (WFP) provides food rations and implementing partners, usually non-government organizations (NGOs), implement nutrition and health programs with support from UNHCR and WFP.

Routine monitoring data is available from many of these programs, however operational challenges, varied reporting structures, and lack of coordination across implementing agencies has limited the utility of this information for the assessment of changes in population health status or comparisons of trends across regions. Often, the most useful information concerning the nutritional status of refugee populations is collected through periodic nutrition surveys that can be of variable quality [9]. In 2005, UNHCR launched a health information system (HIS) to enhance the quality and consistency of routine health information available in protracted refugee situations. The HIS was initially piloted in three countries in East Africa (Tanzania, Kenya, and Ethiopia) and is now operational over 90 refugee camps in 18 countries worldwide where it used to monitor health and nutrition services provided to more than 1.5 million refugees by UNHCR and their partners [10] This paper reviews the nutrition indicators collected in the HIS and examines the extent to which they can be used to monitor and evaluate the performance of UNHCR's nutrition programs in the field. It is the first global analysis of refugee nutrition programs using a standardized routine data source.

## Methods

### UNHCR Nutrition Programs

UNHCR standards state the global acute malnutrition (GAM) should not exceed 10% of the under-five population. All children under five years in UNHCR camps should participate in routine growth monitoring as part of the Expanded Program for Immunizations (EPI), which is managed by the maternal and child health (MCH) unit at the main camp health facility. UNHCR guidelines recommend for monthly growth monitoring visits for children under five, with quarterly visits as a minimum standard [11]. Children identified as underweight, with weight-for-age measures falling in the 60-80% median range or < 60% median on the National Center for Health Statistics/World Health Organization (NCHS/WHO) growth curve^b ^are referred to supplementary and therapeutic feeding centers for further evaluation. Feeding program admission criteria are defined by nutritional status. Children under five with moderate acute malnutrition (MAM), defined by weight-for-height of 70% to 80% of the NCHS/WHO median are enrolled in supplementary feeding programs (SFP) that provide additional weekly rations until weight increases to 90% of the NCHS/WHO median at two consecutive weekly weighings. Upon SFP exit, length of stay for children under five is calculated in number of weeks in the program. UNHCR indicators for well performing SFP include length of stay less than 8 weeks, recovery rate higher than 75%, default rate less than15% and death rate less than 3%. Children under five with severe acute malnutrition (SAM), defined as weight-for-height < 70% of the NCHS/WHO median or oedema, are admitted to in-patient or daily therapeutic feeding programs (TFP) for stabilization. Children admitted for severe acute malnutrition are discharged from TFP to SFP weight-for-height stabilizes at 80% of the NCHS/WHO median or oedema symptoms disappear [12]. Upon TFP exit, length of stay (days) and average weight gain (g/kg/day) are calculated. UNHCR standards for well performing TFP include length of stay less than 30 days, weight gain > 8 g/kg/day, recovery rate higher than 75%, default rate less than 15% and death rate less than 10%. Program enrollment refers to the number of children participating in SFP or TFP at the end of the reporting month. Program coverage refers to the proportion of children under-five with MAM enrolled in SFP or proportion of children under-five with SAM enrolled in TFP.

### Data Analysis

Monthly HIS data on camp population, EPI growth monitoring participation, and nutrition programs were exported from the UNHCR's HIS database [13]. Nutrition program data included the number of children admitted to feeding programs, number remaining enrolled in the program at the end of each month, mean length of stay, reason for discharge (recovery, referral, drop-out), and average weight gain (TFP only). Information on nutrition program admission and enrollment was combined with population data to calculate admission rates and proportions.

Inclusion criteria developed for the analysis of HIS nutrition data included: 1) time period of reporting--the HIS was piloted in 2005 and implemented more broadly in subsequent years; analysis was limited to the period January 2007 to May 2009 to ensure the data were more representative of the entire UNHCR refugee camp population; 2) low or no reporting--camps were omitted to limit inconsistencies during aggregation if they reported data for less than six months or reported less than one percent of the under-five population enrolled in SFP or TFP (these included Cameroon and Guinea for SFP, and Burundi, Cameroon, Guinea, Nepal, Yemen, and Zambia for TFP); and 3) admission criteria--analysis was limited to children under five that were admitted for malnutrition. Microsoft Excel was used for visual data exploration and to create tables and figures for publication. Descriptive statistics (frequency, mean, median, range) and odds of admission/enrollment were calculated using STATA 11. Because the focus of the analysis is program evaluation, camps were weighted equally, regardless of population size.

## Results

**Figure 1:**
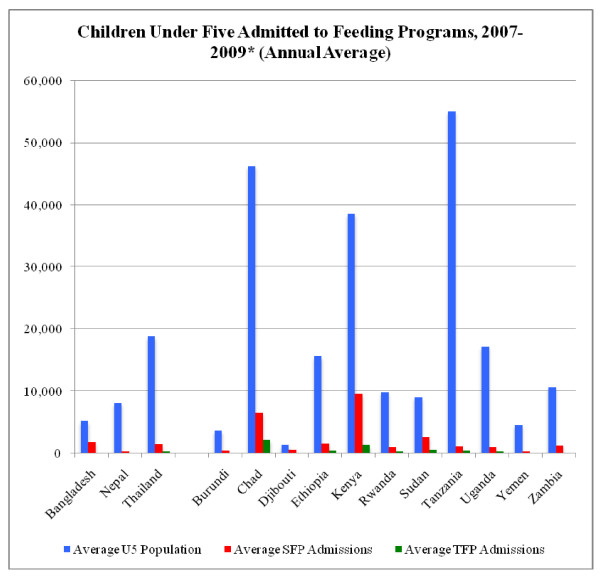
**Average under five refugee population by country, 2007 to mid-year 2009**.

Camp populations covered by the HIS remained relatively constant between 2007 and mid-2009 with averages of 192,472 under five children covered in 2007 (31%), 218,873 covered in 2008 (36%), and 206,441 in 2009 (33%). When proportion of under five children covered by the HIS was assessed by region, 87% were in Africa and 13% in Asia; under five refugee populations by country are provided in Figure 1. When growth monitoring was evaluated in terms of utilization rates, Asia had comparatively high utilization at 60% as compared to 34% in Africa. The proportion of children identified in growth monitoring as having acute malnutrition or wasting, which is indicated by low weight-for-height, is not recorded in the HIS; instead, acute malnutrition prevalence from camp-level nutrition surveys is entered in the HIS intermittently. The average MAM and SAM rates reported in 74 of 81 camps for the 2007-2009 time periods were 7.0% and 1.6%, respectively. Country level MAM rates ranged from 1.1% in Zambia (2008) to 15.8% in Sudan^a ^(2009) and SAM rates ranged from 0.1% in Tanzania (2008) to 5.0% in Chad (2009).

### Supplementary feeding programs

There were a total 57,005 SFP admissions between January 2007 and May 2009 and more than half of admissions occurred in 2008. Under five refugee SFP admissions and enrollment are summarized by year, continent, and country in Table 1; camp level SFP and TFP enrollments are summarized in Table 2. The overall SFP admission rate was 151/1000/yr (range 1-540). Children in Asia accounted for 7% of SFP admissions and were significantly less likely to be admitted to SFP than children in Africa (OR = 0.41, CI: 0.39-0.42). However, when assessed at the country level, average SFP admission and enrollment rates were higher in Asia than in Africa due to high admission and enrollment rates in Bangladesh and the small number of Asian countries reporting. Re-admissions, defined as malnourished children enrolling in SFP within one month of successful discharge, accounted for 8.6% of total admissions, and were substantially higher in Asia (31.9%) as compared to Africa (6.3%). The lowest re-admissions levels were observed in Zambia (2.0%), Tanzania (2.1%) and Ethiopia (4.3%).

**Table 1:** Beneficiary population and SFP feeding program admissions by year, region, and country

	Under Five Population		Supplementary Feeding Program							Moderate Acute Malnutrition Prevalence**
			
			*Admissions (# admitted monthly)*				*Total Enrollment (# enrolled monthly)*			
	Average	% of total	Average new admissions	% of new admissions	Admission Rate (per 1000/yr)	Odds of Admission (CI)*	Average Enrollment	% of Children Enrolled	Odds of Enrollment (CI)	
***By Year***										
2007	192,472	31.2%	25,694	23.8%	74	0.49 (0.36-0.67)	6,635	4.6%	0.66 (0.63-0.68)	***---***
2008	218,873	35.4%	27,678	53.1%	122	0.85 (0.65-1.11)	9,631	5.7%	0.85 (0.83-0.88)	***---***
2009*	206,441	33.4%	12,016	23.1%	140	Reference	10,598	5.2%	Reference	***---***

***By Region***										
**Africa**	**211,336**	**86.9%**	**413**	**81.5%**	**148**	**Reference**	**1474**	**0.7%**	**Reference**	***---***
Burundi	3,651	1.5%	8	1.6%	100	0.64 (0.48-8.45)	23	2.0%	2.94 (2.29-3.73)	**4.2%**
Chad	46,234	19.0%	37	7.3%	140	0.94 (0.72-1.21)	101	3.3%	4.86 (4.51-5.22)	**12.3%**
Djibouti	1,283	0.5%	40	7.9%	397	3.79 (3.04-4.74)	158	12.4%	20.14 (16.86-24.05)	**12.7%**
Ethiopia	15,535	6.4%	18	3.6%	98	0.63 (0.47-0.83)	89	4.5%	6.67 (6.08-7.33)	**9.8%**
Kenya	38,588	15.9%	195	38.5%	248	0.24 (0.21-0.29)	587	6.6%	10.03 (9.39-10.71)	**11.3%**
Rwanda	9,817	4.0%	25	4.9%	99	0.63 (0.47-0.83)	96	3.1%	4.57 (4.02-5.18)	**6.9%**
Sudan	9,012	3.7%	30	5.9%	291	2.36 (1.88-2.97)	194	13.7%	22.63 (20.89-24.51)	**17.9%**
Tanzania	38,588	15.9%	10	2.0%	19	0.11 (0.65-0.18)	26	0.5%	0.75 (0.64-0.87)	**2.3%**
Uganda	17,121	7.0%	11	2.2%	59	0.36 (0.26-0.50)	25	1.2%	1.72 (1.47-1.99)	**4.3%**
Yemen	4,518	1.9%	14	2.8%	62	0.38 (0.27-0.52)	42	1.6%	2.24 (1.73-2.85)	**N/A**
Zambia	10,591	4.4%	25	4.9%	117	0.76 (0.58-0.99)	133	5.0%	7.47 (6.74-8.28)	**6.2%**

**Asia**	**31,971**	**13.1%**	**94**	**18.5%**	**171**	**Reference**	**302**	**3.3%**	**Reference**	***---***
Bangladesh	5,148	2.1%	77	15.2%	351	2.62 (2.11-3.25)	223	8.6%	2.77 (2.47-3.12)	**8.6%**
Nepal	8,082	3.3%	3	0.6%	29	0.14 (0.09-0.21)	29	2.3%	0.70 (0.59-0.82)	**10.5%**
Thailand	18,741	7.7%	14	2.8%	77	0.40 (0.30-0.54)	50	2.7%	0.83 (0.74-0.92)	**2.7%**

***OVERALL***	***243,307***	***100.0%***	***507***	***100.0%***	***151***	***---***	***1,776***	***3.80%***	***---***	***---***

*Includes January thru May 2009 only.

**Prevalence as reported in UNHCR 2008 Public Health Factsheets.

**Table 2:** Camp Level TFP and SFP enrollment rates by region

*TFP Enrollment Rate*	*SFP Enrollment Rate*			
	
	*Low (< 2.5%)*	*Medium (2.5-4.9%)*	*High (5.0-9.9%)*	*Very High (10+%)*
***ASIA***				

Very Low (<.25%)	Nu Poh (Thailand)	Mae La Oon (Thailand)	Ban Don Yang (Thailand)	
	Mae La (Thailand)			
	Mae Ra Ma Luang (Thailand)			
	Umpiem Mai (Thailand)			

Medium (0.25-.49%)			Kutupalong (Bangladesh)	
			Nayapara (Bangladesh)	

High (0.5-0.9%)				

Very High (> 1%)				

***AFRICA***				

Very Low (<.25%)	Dimma (Ethiopia)	Djabal (Chad)	Oure Cassoni (Chad)	Shimelba (Ethiopia)
	Lugufu (Tanzania)	Gaga (Chad)	Hagadera (Kenya)	
	Lugufu II (Tanzania)	Goz Amer (Chad)	Dagahaley (Kenya)	
	Lukole (Tanzania)	Kounoungou (Chad)		
	Mtabila(Tanzania)	Yaroungou (Chad)		
	Nyarugusu (Tanzania)	Bonga (Ethiopia)		
	Nduta (Tanzania)	Kebribeyah (Ethiopia)		
	Kyaka II (Uganda)	Kakuma (Kenya)		
	Kyangwali (Uganda)			
	Nakivale (Uganda)			

Medium (0.25-.49%)	Amboko Chad)	Dosseye (Chad)	Dosseye (Chad)	Kilo 26 (Sudan)
	Kiziba (Rwanda)	Bredjing (Chad)	Ifo (Kenya)	Wad Sharifey (Sudan)
		Fugnido (Ethiopia)		
		Sherkole (Ethiopia)		
		Kanembwa (Kenya)		
		Nyabiheke (Rwanda)		

High (0.5-0.9%)	Oruchinga (Uganda)	Gihembe (Rwanda)	Awbarre (Ethiopia)	Girba (Sudan)
				Shagarab I II III (Sudan)

Very High (> 1%)		Kiryandongo (Uganda)	Suki (Sudan)	Abuda (Sudan)
			Ikafe (Uganda)	Fau 5 (Sudan)
				Um Gargour (Sudan)

Note: TFP enrollment categories for camps excluded from the SFP analysis were as follows: Low--Adjumani and Palorinya (Uganda); Medium--Kanembwa (Uganda); Very High--Ikafe (Uganda). SFP enrollment categories for camps excluded from the TFP analysis were as follows:Low--Mtabila II, Nduta, and Kanembwa (Tanzania); Khudunabari, Beldangi II Ext, Timai, and Goldhap (Nepal); Kyangwali and Madi Okollo (Uganda); Dimma (Ethiopia), and Kouankan II (Guinea); Medium--Beldangi I & II and Sanishare (Nepal); Amnaba, Gondje, Treguine, and Gaga (Chad), and Bonga (Ethiopia); High--Oure Cassoni (Chad); and Very High--Ali Adde (Djibouti) and Shimelba (Ethiopia).

SFP entry and exit criteria are variable and many SFP programs target the malnourished in addition to other vulnerable groups. Average monthly enrollment for MAM children in SFP programs (Table 1) provides a better perspective on program size and coverage of the under-five population. Country-level SFP admissions ranged from a low of 16/1000/yr in Tanzania to a high of 397/1000/yr in Djibouti. Bangladesh, Sudan and Kenya also had notably high SFP admission rates. Although the country-level admission rate was highest in Djibouti at 14.6%, the refugee population in Sudan, with a 12.4% enrollment rate, is seven times larger and contributed substantially higher number of enrollment. Kenya also had large numbers of children enrolled in SFPs with an average enrollment rate of 9.1% of children under five. On average, 4.7% (range of 0.5-13.7%) children less than five years of age in UNHCR refugee camps were enrolled in SFPs because of poor nutritional status.

SFP performance consistently exceeded all UNHCR standards with the exception of length of enrollment (Table 3). Average length of SFP enrollment was 12 weeks in Africa and 22 weeks in Asia as compared to the UNHCR standard of < 8 weeks. All regions had average recovery rates above 80% (standard > 75%), death rates below 1% (standard < 3%) and default rates below 9% (standard < 15%). At the country level, only Thailand (70%) and Yemen (55%) did not meet the > 75% standard for exits due to recovery. Thailand's default and death rates did fall within the standards; Yemen had a default rate of 28%, which was nearly double the UNHCR standard. In addition, nine camps in other countries (Chad, Nepal, Rwanda, Tanzania and Zambia) did not meet the UNHCR standard for recovery. All countries fell within the acceptable mortality rate standard of < 3% with the exception of Zambia where on average, SFPs had a 5.7% mortality rate. Outside of Zambia, mortality rates ranged from 0% in many camps to 3.8% in Farchana camp in Chad and 5.4% in Kilo 26 camp in Sudan.

**Table 3:** SFP Indicators and Program Performance by Region

SFP Indicator	Type	Standard	Africa			Asia		
			**Mean**	**Median**	**Range**	**Mean**	**Median**	**Range**

**Total Admissions (January 2007-May 2009)**	Process	NA		48,630		3,467		

**Length of Stay**	Outcome	< 8 weeks	12.2	8.3	0-132	22.1	12	0-151

**Recovery Rate**	Outcome	> 75%	87.2%	97.0%	8-100%	81.8%	100%	3-100%

**Death Rate**	Outcome	< 3%	0.5%	0	0-33%	0.8%	0	0-50%

**Default Rate**	Outcome	< 15%	7.0%	0	0-85%	2.6%	0	0-60%

### Therapeutic feeding programs

**Figure 2:**
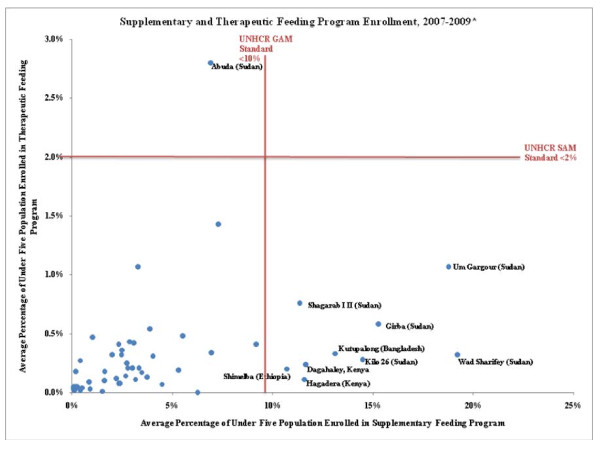
**Average Feeding Program Enrollment by Camp**.

The average TFP admission and enrollment rates were 22/1000/yr (range 0-124) and 5/1000/yr (range 0-45), respectively (Table 4). Figure 2 illustrates the relationship between average SFP and TFP enrollment at the camp level. The majority of camps that have average SFP enrollment rates above 10% have relatively low TFP enrollment rates (≤0.5%) Under five refugee TFP admissions and enrollment are summarized by year, continent, country, and admission type in Table 4. Overall, 79% of TFP admissions were for acute wasting (n = 3,372) and 21% were for oedema (n = 944). Average admission rates were 17/1000/yr for acute wasting and 5/1000/yr for Oedema. The vast majority of TFP admissions were in Africa, with only 5% of admissions reported in Asia (including 7.1% of acute wasting and 0.6% of oedema of admissions). Overall children in Africa were 1.85 (CI: 1.62-2.10) times more likely to be admitted to TFP than children in Asia. Average monthly enrollment for SAM children in TFP programs was less than 1% of the under-five population in most camps; only Sudan and Uganda had camps with higher enrollment. Overall, 6.2% of acute wasting admissions and 3.9% of oedema admissions were re-admissions, and re-admission rates were higher in Africa than Asia. Re-admissions accounted for 20% of acute wasting admissions in Asia and 5% in Africa. For oedema, the re-admission rate was 3.9% in African countries. No re-admissions were reported for oedema in Asia.

**Table 4:** Beneficiary population and TFP feeding program admissions by year, region, country, and admission type

	Under Five Population		Therapeutic Feeding Program							Severe Acute Malnutrition Prevalence **
			
			*Admissions (# admitted monthly)*				*Total Enrollment (# enrolled monthly)*			
	Average	% of total	Average new admissions	% of new admissions	Admission Rate (per 1000/yr)	Odds of Admission (CI)*	Average Enrollment	% of Children Enrolled	Enrollment Odds (CI)	
***By Year***										
2007	185,613	33.6%	2,144	49.7%	8	2.24 (2.07-2.43)	320	0.2%	2.22 (1.81-2.73)	*---*
2008	189,858	34.3%	1,789	41.5%	6	1.83 (1.69-1.98)	258	0.1%	1.74 (1.41-2.16)	*---*
2009*	177,340	32.1%	383	8.9%	5	Reference	138	0.1%	Reference	*---*

***By Region***										
**Africa**	**180,528**	**90.2%**	**321**	**91.7%**	**21**	**Reference**	**371**	**0.2%**	**Reference**	*---*
Chad	32,561	16.3%	102	29.1%	46	2.25 (1.30-3.99)	70	0.2%	1.02 (0.77-1.32)	0.8%
Ethiopia	16,362	8.2%	32	9.1%	23	1.09 (0.57-2.09)	45	0.3%	1.31 (0.93-1.79)	0.7%
Kenya	35,368	17.7%	95	27.1%	35	1.69 (0.95-3.08)	71	0.2%	1.07 (0.83-1.37)	1.3%
Rwanda	9,571	4.8%	18	5.1%	25	1.19 (0.64-2.26)	40	0.5%	2.35 (1.69-3.19)	1.7%
Sudan	8,888	4.4%	28	8.0%	52	2.56 (1.59-4.50)	69	1.6%	7.77 (6.35-9.49)	2.0%
Tanzania	49,746	24.9%	21	6.0%	7	0.33 (0.12-0.81)	32	0.1%	0.39 (0.27-0.54)	0.1%
Uganda	28,032	14.0%	25	7.1%	14	0.66 (0.31-1.37)	44	0.3%	1.62 (1.27-2.03)	2.6%

**Asia**	**19,571**	**9.8%**	**30**	**8.6%**	**26**	**Reference**	**20**	**0.2%**	**Reference**	*---*
Bangladesh	5,050	2.5%	16	4.6%	37	1.44 (0.84-2.49)	10	0.4%	2.11 (1.14-3.79)	0.3%
Thailand	14,521	7.3%	14	4.0%	13	0.49 (0.23-1.00)	10	0.1%	0.50 (0.24-0.96)	0.1%

***By Type***										
Acute wasting	***---***	***---***	3,372	78.1%	17	3.62 (3.36-3.89)	230	0.1%	1.27 (1.04-1.55)	*---*
Oedema	***---***	***---***	944	21.9%	5	Reference	181	0.1%	Reference	*---*

***OVERALL***	***200,099***	***100%***	***351***	***100.0%***	***22***	***---***	***391***	***0.2%***	***---***	*---*

*Includes only January thru May 2009.

**Prevalence as reported in UNHCR 2008 Public Health Factsheets.

TFP performance for acute wasting admissions met UNHCR standards with the exception of daily weight gain in both Africa and Asia and recovery rate Asia (Table 5). Average weight gain for acute wasting admissions in Asia and Africa were 7 g/kg/day and 7.5 g/kg/day, respectively, which fell below the UNHCR standard of > 8 g/kg/day. In Asia, the average recovery rate was below the 75% standard in both Bangladesh (69%) and Thailand (57%), contributing to the low regional average of 66%. In Africa, Kenya and Djibouti, had sub-standard recovery rates of 57% and 70%, respectively; the low recovery rate in Djibouti was attributed to a high default rate. TFP performance for oedema admissions met all UNHCR standards with the exception of recovery and death rates in Asia (Table 6). In Asia, the average recovery rate was 50% (UNHCR standard > 75%) and the average mortality rate was 17% (UNHCR standard < 10%). High oedema mortality and low recovery rates in Asia is likely due to a 50% average mortality rate in Mae La camp and no reporting from other camps in Thailand. No oedema deaths were reported in Bangladesh.

**Table 5:** TFP Indicators and Program Performance for Acute Wasting by Region

TFP Indicator	Type	Standard	Africa			Asia		
			
			Mean	Median	Range	Mean	Median	Range
**Total acute wasting admissions (January 2007-May 2009)**	Process	N/A		3,171			201	

**Length of Stay**	Outcome	< 30 days	27.2	22.7	0-600	29.9	28.9	4-82

**Weight gain**	Outcome	> 8 g/kg/day	7.5	7.7	0-32	7.0	5	0-46

**Recovery Rate**	Outcome	> 75%	78.9%	100%	0-100%	66.3%	83%	0-100%

**Death Rate**	Outcome	< 10%	4.6%	0%	0-100%	5.9%	0%	0-100%

**Default Rate**	Outcome	< 15%	7.3%	0%	0-100%	9.0%	0%	0-100%

**Table 6:** TFP Indicators and Program Performance for Oedema by Region

TFP Indicator	Type	Standard	Africa			Asia		
			
			Mean	Median	Range	Mean	Median	Range
**Total oedema admissions (January 2007-May 2009)**	Process	N/A		938			6	

**Length of Stay**	Outcome	< 30 days	27.2	23.5	0-352	21.7	25	14-26

**Weight gain**	Outcome	> 8 g/kg/day	12.0	8	0-368	20.7	6	16953

**Recovery Rate**	Outcome	> 75%	78.4%	100%	0-100%	50.0%	50%	6-50

**Death Rate**	Outcome	< 10%	5.7%	0%	0-100%	16.6%	0%	0-100%

**Default Rate**	Outcome	< 15%	3.7%	0%	0-100%	0%	0%	0%

## Discussion

SFP and TFP admission rates were consistently higher in Africa than in Asia. Most refugee camp populations receive rations that account for most of their nutritional needs. However, the total ration amount provided frequently depends on access to markets, livelihood opportunities, donor funding, and supply chain logistics. Children in camps in Africa were significantly more likely to be admitted to SFPs and TFPs than those in Asia due to more unstable situations in Africa (e.g. insecurity and population movements in numerous camps due to conflict). Furthermore, unlike in Asia, many of the camps in Africa are situated in arid conditions where small scale agricultural projects are more difficult to implement.

SFP performance consistently met all standards with the exception of length of enrollment (standard of < 8 weeks), which was exceeded in 79% of camps reporting. UNHCR's SFPs predominantly provide dry weekly take home rations for MAM children and their households. Slow recovery rates may be a function of children not consuming adequate amounts of the supplementary ration. Sharing of dry take home rations is a frequent challenge in SFP programs; even when additional food is provided for other household members it can be difficult to ensure that the targeted child receives adequate amounts of the provided supplementary foods [14,15] In situations where ration reductions have occurred or where market conditions and/or population preferences favor the sale of rations, extended SFP enrollment periods may be especially problematic. In certain contexts where failure to meet length enrollment standards is of particular concern, wet feeding programs or ready-to-use foods may promote faster recovery (and thus shorter enrollment). However, these approaches to supplementary feeding can be time and cost intensive [16,17].

SFPs in all countries fell within the acceptable mortality standard of < 3% with the exception of Zambia which had a 5.7% mortality rate. Overall, 4% (n = 4) of camps did not meet the SFP mortality standard including 2 of 4 camps in Zambia. GAM prevalence among children in Zambia was estimated at 6.2% in December 2008, which is above the UNHCR standard of 5% for that time period (the standard has since been changed to 10%). However, the under-five mortality rate was reportedly relatively low at 5 deaths/1,000 under-5 children/year [18].

Underweight status is associated with increased risk of infectious disease morbidity and childhood mortality [19,20]. Malaria accounted for more than one third of morbidity in Zambia refugee camps, and an estimated 57% of malarial illness in children is attributed to underweight status. Children that are moderately and severely underweight are five to eight times as likely to die before the age of five as compared to children that were better nourished [21]. Thus it is likely that children in MAM programs would have elevated mortality rates, though this would not be limited to Zambia and the highest mortality rates would be anticipated among children in TFPs.

Overall, 94% of TFP admissions were in Africa and children in Africa were significantly more likely to be admitted to TFP than those in Asia. Access to food in African camps appears to be a greater concern than in Asian camps, however, effective SFP and growth monitoring programs with high coverage levels would ideally reduce the number of TFP admissions in food insecure contexts. The fact that majority of camps that have average SFP enrollment rates above 10% have relatively low TFP enrollment rates (≤0.5%) suggests that growth screening and SFP objectives are being met and that SAM is relatively well controlled. Camps with both high SFP and TFP enrollment rates (Um Gargour, Suki, and Abuda, all in Sudan), however, are deserving of additional attention because this is potentially indicative of a poor nutrition situation. SFP enrollment in most camps in Sudan exceeds 10% and Um Gargour and Wad Sharifey had especially high SFP enrollment rates at 19%; the situation in Abuda appears particularly critical, where the TFP enrollment rate of 2.8% exceed the UNHCR standard for SAM of < 2%.

Camp level statistics for Sudan were not available however, aggregated data for all camps indicates generally poor nutritional status among refugees, with GAM and SAM rates of 17.1% and 2.1%, respectively, and inadequate daily ration of 1575 kcal/person/day [18]. Increasing the general ration, blanket supplementary feeding in camps where GAM exceeds 15%, and active screening for malnutrition which would result in increased SFP and TFP enrollment are steps that could be taken to lower malnutrition prevalence rates and stabilize the nutrition situation [22].

**Figure 3:**
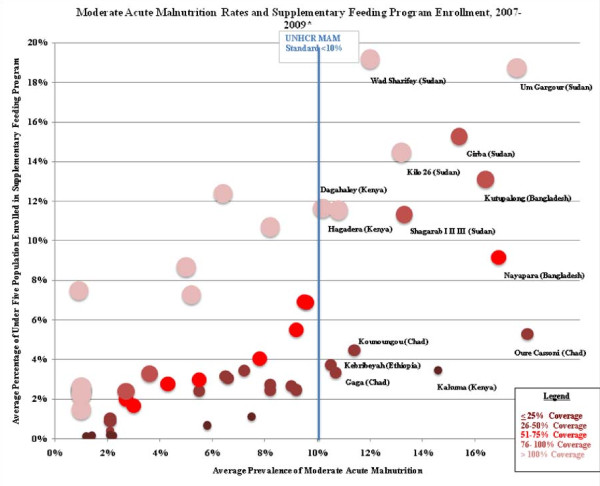
**Global Acute Malnutrition Rates and Supplementary Feeding Program**. Enrollment. *MAM prevalence was calculated as the difference between GAM and SAM prevalence from 2008 surveys reported in UNHCR Nutrition Survey database or most recent year where 2008 data not available; all prevalence data measured using WHO growth standards except for Gasorwe and Musasa (Burundi), Awbarre and Kebribeyah (Ethiopia), Wad Sharifey (Sudan), and Basteen and Kharaz (Yemen) which only reported GAM and SAM prevalence using NCHS growth standards. Note: GAM and SAM prevalence were measured across multiple camps in a joint survey in Amboko & Gondje (Chad); Adjumani, Impevi, Kiryandongo, Palorinya & Rhino (Uganda) and Kyaka, Kyangwali, Nakivale, & Oruchinga (Uganda).

**Figure 4:**
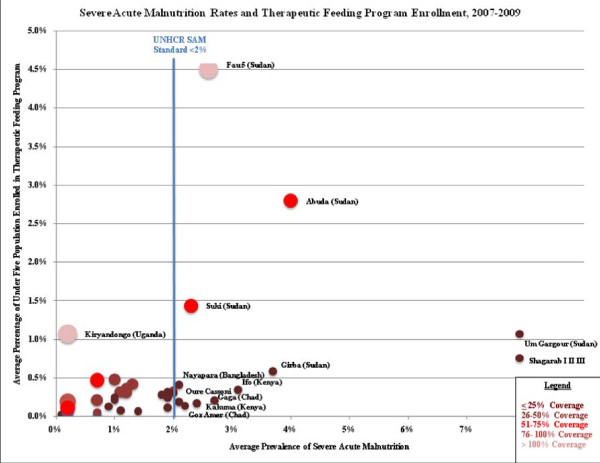
**Severe Acute Malnutrition Rates and Supplementary Feeding Program**. Enrollment * SAM prevalence from 2008 surveys reported in UNHCR Nutrition Survey database or most recent year where 2008 data not available; all prevalence data measured using WHO growth standards except for Awbarre, Kebribeyah, Shimelba (Ethiopia) and Wad Sharifey (Sudan) which only reported SAM prevalence using NCHS growth standards. *Note: *SAM prevalence was measured across multiple camps in a joint survey in Amboko & Gondje (Chad); Adjumani, Impevi, Kiryandongo, Palorinya & Rhino (Uganda) and Kyaka, Kyangwali, Nakivale, & Oruchinga (Uganda).

Camp-level MAM prevalence, SFP enrollment rates, and estimated SFP coverage rates are presented in Figure 3. SFP coverage rates varied widely, however, nearly half of the camps (22 of 53 where MAM could be calculated) were estimated to fall below 50% which is concerning. It is possible that coverage estimates are inaccurate due to problems with surveys where MAM prevalence was over estimated or due to unreliable population denominator estimates. Another potential scenario is that poor coverage and use of growth monitoring programs and lack of active nutrition screening programs result in low identification and referral rates of MAM children to SFP programs. Camp-level SAM prevalence, TFP enrollment rates, and estimated TFP coverage rates are shown in Figure 4. SFP coverage rates varied widely in the majority of camps (9 of 12) where SAM rates exceeded the UNHCR standard of 2%, TFP coverage rates were estimated at < 25%. As with MAM, it is possible that coverage rates were underestimated as a result of poor quality nutrition survey data, however it is unlikely that this alone is would result in consistently low coverage rates. Active surveillance for MAM and SAM and subsequent referral of malnourished children to SFP and TFP programs is a critical activity that could increase the coverage and effectiveness of UNHCR nutrition programs.

TFP performance was variable when compared to UNHCR standards, suggesting the need for targeted improvement efforts in some camps. TFP readmission rates in Africa were more than four times higher than those in Asia for both acute wasting and oedema. This suggests that SFP programs in camps with high TFP admission and re-admission rates should be examined for potential improvements. In addition, camps with small SFP programs but high TFP enrollment (Kiryandongo and Oruchinga in Uganda, Gihembe in Rwanda) could expand and improve SFP programming to prevent deterioration from MAM to SAM. The ongoing transition to community-based therapeutic care (CTC) for uncomplicated SAM cases may be effective in reducing TFP re-admission rates. The TFP length of stay standard (< 30 days) was frequently exceeded, however, regional averages for length of stay only exceed this standard by several days and this indicator may not be of particular importance provided that daily weight gain standards are met.

Other TFP program performance measures indicate a need for close monitoring during the transition to CTC. This is particularly true in Asia where low recovery rates, substandard weight gain, high oedema death rates observed. However, given that Asia comprises only 5% of TFP admissions, a focus on improving TFP performance in Africa would benefit a larger population. Given that 54.7% of SFP beneficiaries and 33.6% of TFP beneficiaries worldwide are in Kenya, feeding program improvements here would yield sizeable benefits in terms of absolute reductions in malnutrition prevalence and mortality rates among refugee children of concern to UNHCR.

### Limitations

The primary aim of the HIS is to provide basic information on refugee health status and services provided by health facilities in camps. There are several key limitations when trying to draw conclusions related to nutritional status. Firstly, as HIS data is predominantly collected in health facilities, it may be biased because populations that do not seek care are excluded. As camps are small, circumscribed areas and their residents generally do not access outside health services, this is not perceived as a major concern. Secondly, because data are reported at camp level and not at the individual level, some information cannot be gleaned from available data. For example, it would have been useful to assess individual predictors for feeding program enrollment or re-admission but this is rarely possible from routine nutritional surveillance. Thirdly, the frequency of reporting was sometimes inconsistent or implausible monthly variations were observed in some reported values. Outliers were dropped and averages were used to minimize the effect of inconsistencies on findings, however, the overall quality of nutrition data used was variable. Similar findings were noted in a recent Centers for Disease Control (CDC) evaluation of the HIS [23,24]. Finally, information was lacking on several key indicators that could have greatly contributed to analysis; these included growth monitoring outcomes, prevalence of malnutrition (necessary to calculate coverage rates), information on ration content and frequency of distributions, and the extent and use of community-based therapeutic care (CTC) programs for SAM children which is beginning to replace facility-based programs and which was reported only for a minority of camps.

## Conclusions

UNHCR's HIS is a primary source of routine feeding program data collected using standardized case definitions and reporting formats across refugee camps in multiple settings. Findings from this paper, which analyzed available data from growth monitoring, supplementary and therapeutic feeding programs, includes more than 90 refugee camps in 18 countries and provides the first comprehensive assessment of feeding programs in UNHCR refugee camps worldwide. A number of important findings with regard to the performance of selective feeding programs in post-emergency settings were identified in addition to areas requiring further analysis and programmatic improvements. Higher growth monitoring coverage rates (≥ 90%, the UNHCR standard) of children under-five or active surveillance for malnourished children would increase the ability of UNHCR and its partners to identify and treat cases of acute malnutrition. Additionally the inclusion of the number of children identified as moderately and severely wasted would allow UNHCR to better track and respond to changes in acute malnutrition prevalence rates. Expansion of nutrition reporting in the HIS will be especially important during the transition to community-based therapeutic care if CTC performance is to be adequately monitored. In terms of priority regions for program improvement, a focus on camps and countries with large refugee populations and high feeding program enrollment rates, in particular Kenya and Sudan, would have the greatest impact in terms of absolute reductions in the incidence and prevalence of malnutrition among children in UNHCR refugee camps.

## Authors' contributions

SD conceived of the study and led the manuscript drafting and finalization process. HT led data analysis and assisted with drafting the manuscript. CH provided HIS technical support for data analysis and critical review of the manuscript. CW contributed to drafting and critical review of the manuscript. PS conceived of the study and contributed to critical review of the manuscript. All authors read and approved the final version of the manuscript.

## Funding

This study was supported by the United Nations High Commissioner for Refugees.

## Competing interest declaration

The authors declare that they have no competing interests.

## Endnotes

^a ^In the case of Sudan, country-level analysis refers only to the post-emergency camps in East Sudan.

^b ^UNHCR has since transitioned to the use of the 2006 WHO International Reference Population for many of its programs.
